# Cluster analysis in severe emphysema subjects using phenotype and genotype data: an exploratory investigation

**DOI:** 10.1186/1465-9921-11-30

**Published:** 2010-03-16

**Authors:** Michael H Cho, George R Washko, Thomas J Hoffmann, Gerard J Criner, Eric A Hoffman, Fernando J Martinez, Nan Laird, John J Reilly, Edwin K Silverman

**Affiliations:** 1 Channing Laboratory, Brigham & Women's Hospital, Boston, MA, USA; 2 Division of Pulmonary and Critical Care Medicine, Brigham & Women's Hospital, Boston, MA, USA; 3 Department of Biostatistics, Harvard School of Public Health, Boston, MA, USA; 4 Division of Pulmonary and Critical Care, Temple University School of Medicine, Philadelphia, PA, USA; 5 Department of Radiology, Carver College of Medicine, University of Iowa, Iowa City, IA, USA; 6 Division of Pulmonary and Critical Care Medicine, University of Michigan Health System, Ann Arbor, MI, USA; 7 University of Pittsburgh Medical Center, Pittsburgh, PA, USA

## Abstract

**Background:**

Numerous studies have demonstrated associations between genetic markers and COPD, but results have been inconsistent. One reason may be heterogeneity in disease definition. Unsupervised learning approaches may assist in understanding disease heterogeneity.

**Methods:**

We selected 31 phenotypic variables and 12 SNPs from five candidate genes in 308 subjects in the National Emphysema Treatment Trial (NETT) Genetics Ancillary Study cohort. We used factor analysis to select a subset of phenotypic variables, and then used cluster analysis to identify subtypes of severe emphysema. We examined the phenotypic and genotypic characteristics of each cluster.

**Results:**

We identified six factors accounting for 75% of the shared variability among our initial phenotypic variables. We selected four phenotypic variables from these factors for cluster analysis: 1) post-bronchodilator FEV_1 _percent predicted, 2) percent bronchodilator responsiveness, and quantitative CT measurements of 3) apical emphysema and 4) airway wall thickness. K-means cluster analysis revealed four clusters, though separation between clusters was modest: 1) emphysema predominant, 2) bronchodilator responsive, with higher FEV_1_; 3) discordant, with a lower FEV_1 _despite less severe emphysema and lower airway wall thickness, and 4) airway predominant. Of the genotypes examined, membership in cluster 1 (emphysema-predominant) was associated with *TGFB1 *SNP rs1800470.

**Conclusions:**

Cluster analysis may identify meaningful disease subtypes and/or groups of related phenotypic variables even in a highly selected group of severe emphysema subjects, and may be useful for genetic association studies.

## Background

Chronic Obstructive Pulmonary Disease (COPD) is defined by the Global Initiative for Chronic Obstructive Lung Disease (GOLD) as airflow limitation that is not fully reversible[1]. This deliberately broad and simple definition based on reduced expiratory airflow has been useful in leading to increased awareness and understanding of the disease[2]. However, substantial heterogeneity within this definition exists[3,4]. Moving beyond spirometry and evaluating other variables is critical to understanding differences in patients with COPD, to gain mechanistic insights into the disease, to identify those at highest risk of specific outcomes, and to personalize therapy [4-7].

Substantial evidence indicates that genetic variation contributes to differences in COPD susceptibility; however, replication of genetic associations in COPD - and in many other complex diseases - has generally been poor[8]. Disease heterogeneity is likely an important factor for these inconsistent findings[9,10]. Several attempts to overcome heterogeneity have been used, including using classic subtypes of chronic bronchitis or emphysema[11], defining subtypes based on a pathophysiologic characteristic (such as rapid or slow decline in lung function[12]), or assessing phenotypic characteristics by chest CT scans[13].

Statistical learning techniques offer an opportunity to extract novel patterns and trends from phenotypic data[14], and thus identify COPD subtypes without using a priori expectations about disease characteristics[15-20]. To our knowledge, these strategies have not been applied in a group with severe emphysema, nor have studies used these subtypes in a genetic association study. We hypothesized that cluster analysis would identify distinct subtypes of COPD subjects, and that variants in COPD candidate genes would be associated with these subtypes.

## Methods

Details of subject recruitment and phenotyping in the National Emphysema Treatment Trial (NETT) have been reported previously[21]. Briefly, NETT participants had physician-diagnosed COPD, FEV_1 _≤ 45% predicted, evidence of hyperinflation on pulmonary function testing, and bilateral emphysema on chest CT scan. Enrollment in the NETT Genetics Ancillary Study began after the initiation of the clinical trial, and thus only a subset of the original cohort had DNA available for genotyping. The characteristics of NETT subjects included and excluded from this analysis are shown in Additional File 1, Table S1. Participants gave written informed consent. The appropriate institutional review boards approved all studies. Self-identified white subjects in the NETT Genetics Ancillary Study with complete CT phenotypic data (emphysema and airway wall quantitative measures) were included in the analysis.

We selected a set of 31 CT, lung function, and other key phenotypic variables, based on clinical relevance, inclusion in previous genetic association studies, and complete data, to avoid subject drop-out (Table 1). Measurements of the phenotypic variables have been previously described[21-25]. 12 SNPs from 5 genes were chosen on the basis of available genotyping and prior associations with COPD (Table 2), and included genes involved in xenobiotic metabolism (*EPHX1 *and *GSTP1*) and surfactant homeostasis (*SFTPB*), as well as two genes identified in part through linkage studies: *TGFB1*, a cytokine growth factor, and *SERPINE2*, a thrombin and urokinase inhibitor. A limited number of SNPs were selected in order to limit multiple statistical testing in this relatively small study population[26]. For the gene *SERPINE2*[27,28], in which numerous associations have been described, we chose SNPs tagging associations found in at least two populations, using a r^2 ^cutoff of > 0.8 in Tagger [29] as implemented in Haploview 4.1 [30].

**Table 1:** Factor analysis.

	Factor 1	Factor 2	Factor 3	Factor 4	Factor 5	Factor 6
Age, years	0.226					

BMI, kg/m2		0.272		-0.123	0.117	

Gender (% male)	0.181	-0.119	0.153			-0.712

Pack-years of smoking						0.187

Age started smoking						-0.237

Age quit smoking	0.146	0.14				-0.11

Pre-bronchodilator FEV_1_, % predicted	0.852		0.466		-0.181	0.1

Pre-bronchodilator FVC, % predicted	0.903		-0.247			0.154

Post-bronchodilator FEV_1_, % predicted	0.853		0.461		0.209	

Post-bronchodilator FVC, % predicted	0.899		-0.301		0.172	0.193

Bronchodilator response, % of baseline FEV_1_					0.979	

Bronchodilator response, absolute change in FEV_1_, L	0.163				0.943	0.183

FEV_1_/FVC ratio, post-bronchodilator	0.104		0.961			-0.134

FEV_1_/FVC ratio, pre-bronchodilator			0.902		-0.106	

Total lung capacity, % predicted		-0.165	-0.227	0.151		

Residual volume, % predicted	-0.506		-0.145	0.199	-0.132	

Diffusion capacity, % predicted	0.169		0.219	-0.253		0.286

Total fraction emphysema at -950 HU		-0.214	-0.178	0.822		

Difference between apical and basal emphysema at -950 HU				0.846		-0.108

Apical fraction emphysema at -950 HU		-0.141		0.981		

Airway wall thickness, mm		0.96				

Airway wall area, %		0.94		-0.114		

Square root wall area, cm		0.882				

6 minute walk distance, ft	0.175	-0.105			0.104	0.566

Maximum work, watts	0.174		0.135	-0.1	0.164	0.791

UCSD Shortness of Breath Questionnaire	-0.227					-0.307

Arterial pH	0.212		0.107	0.146		

PaO_2_, mmHg	0.2		0.205			0.256

PaCO_2_, mmHg	-0.293		-0.345	-0.104		-0.166

Exacerbations in year prior to randomization		-0.123				-0.113

Exacerbations/year (over 3.3 years)		-0.107	-0.204			0.152

Six factors were identified accounting for 75% of the common variance. Higher factor loadings indicate higher correlations of the variable with that factor. Loadings ≥ |0.1| are shown.

**Table 2:** Single nucleotide polymorphisms (SNPs).

Gene Symbol	SNP	Major/Minor Allele	Minor Allele Frequency	Association in NETT (Effect of Variant Genotype)	Other Reported COPD Association(s)
*EPHX1*	rs1051740 (Tyr113His)	T/C	0.31	Less maximum work[42]	Associations with discordant directions; meta-analysis with a protective effect of the variant allele[55]
	
	rs2234922 (His139Arg)	A/G	0.19	Decreased risk of COPD [9]; Lesser degree of apical minus basilar emphysema [22]; increased DLCO[42], less maximum work after LVRS[56]	Wild type with variant type rs1051740, associated with lung function decline[12]

*GSTP1*	rs1695 (aka rs947894)	A/G	0.36	Lesser degree of apical and apical minus basilar emphysema [22]	Associations with discordant directions [57]

*SERPINE2*	rs6734100	C/G	0.15		Case-control and family-based; variant less common in cases[28]
	
	rs6747096	A/G	0.19	Protection from COPD [27]	
	
	rs975278	C/T	0.20	Decreased apical emphysema [22]	Decreased risk of COPD [28]

*SFTPB*	rs1130866 (Thr131Ile)	A/G	0.46	Associated with COPD, in the presence of a gene-by- environment interaction[9]; fewer exacerbations[23]	Associated with COPD [9,58]
	
	rs2118177	T/C	0.35	Fewer exacerbations[23]	
	
	rs2304566	T/C	0.25	Fewer exacerbations[23]	
	
	rs3024791	C/T	0.16	Fewer exacerbations[23]	

*TGFB1*	rs1800470 (aka rs1982073) (Leu10Pro)	A/G	0.39	Decreased risk of COPD [59]; lower FEV_1 _within emphysema subjects[48]; increased apical emphysema[22]; decreased airway wall thickness (unpublished observations)	Decreased risk of COPD[60,61]
	
	rs1800469	G/A	0.30	Decreased risk of COPD[59]; lower FEV_1 _within emphysema subjects[48]; greater dyspnea symptoms [42]; increased apical emphysema[22]	

Twelve SNPs from nine candidate genes were chosen based on available genotyping and previous associations.

We used factor analysis as a guide to determine which COPD phenotypic variables to include in our clustering analysis[31]. Factor analysis is a data reduction technique related to principal component analysis, where shared variability in several observed variables is explained in terms of fewer unobserved variables, called factors. The strength of the relationship between the observed variables and factors can be measured by factor loadings. We used factor analysis in two ways: first, to select variables which represent greater amounts of shared variability; and second, among these variables, to select one representative measurement using a high factor loading to avoid over-weighting correlated COPD characteristics, which could bias a cluster analysis.

The goal of cluster analysis is to assign subjects to groups, where subjects in the same cluster are more similar to each other than they are to subjects in other groups[14]. Similarity is generally defined using a measurement of distance, calculated using the difference between measurements. As numerous clustering methods exist, we evaluated the performance of several clustering algorithms. We chose the best performing clustering technique and cluster number using the silhouette width, a measure of how close each point in one cluster is to points in neighboring clusters. We then examined each variable for differences among the clusters.

## Results

A total of 308 subjects from the NETT Genetics Ancillary Study were included in the analysis. Phenotypic variables selected for inclusion in the factor analysis are shown in Table 1. SNPs included for analysis, along with their minor allele frequencies and previous genetic association studies in COPD (both in the NETT cohort and others) are listed in Table 2. Six factors accounted for 75% of the common variance. Eigenvalues for these factors ranged from 1.7 to 4.9; two additional factors (not shown) had eigenvalues > 1.0. The results of the factor analysis are shown in Table 1. These factors were interpreted as: 1) spirometry (containing pre- and post-bronchodilator FEV_1 _and FVC percent predicted); 2) airway wall thickness (wall thickness, derived square root wall area of a 10 mm internal perimeter airway and derived wall area percent of a 10 mm airway); 3) FEV_1_/FVC ratio; 4) quantitative emphysema severity and distribution (divided into equal thirds by absolute lung height from apex to base), using a cutoff of -950 Hounsfield units; 5) bronchodilator responsiveness; and 6) maximum work and gender. The following variables were chosen as representative based on relatively high factor loadings, accounting for a greater proportion of shared variability: post-bronchodilator FEV_1 _percent predicted (for factors 1 and 3), airway wall thickness (factor 2), apical emphysema (factor 4), and bronchodilator responsiveness (factor 5).

Based on measures of silhouette width, the k-means clustering algorithm using four clusters was found to be optimal for this dataset. This optimal value was low (~0.2) consistent with modest separation of the clusters. A plot of the clusters with each pair of phenotypic variables is shown in Additional file 1, Figure S1. Differences between the selected phenotypic variables by cluster are shown in Table 3. As expected, all selected phenotypic variables selected for use in cluster analysis were significantly different between clusters (P < 10^-3^). Cluster 1 had the greatest degree of emphysema and the least airway wall thickness, as well as a lower FEV_1_. Conversely, cluster 4 had the highest airway wall thickness and the least emphysema, and also had lower bronchodilator responsiveness and FEV_1_. Cluster 2 was a milder subgroup, with the highest FEV_1 _and bronchodilator responsiveness, as well as less emphysema. Cluster 3 also had less emphysema and in addition, less airway wall thickness; however, in contrast to cluster 2, this cluster was more severely affected with the lowest FEV_1 _and bronchodilator responsiveness.

**Table 3:** Baseline Phenotypic Characteristics and Results of Cluster Analysis.

	Cohort	Groupwise Test	Cluster			
			
			1	2	3	4
		
N	308		66	102	88	52
**Phenotypic Variables**						

Post-bronchodilator FEV_1_, % predicted	28.3 (7.33)	2.60 × 10^-28^	26.5*	34.3**	23.9**	26.2*

Bronchodilator response, % of baseline FEV_1_	13.6 (0.12)	5.10 × 10^-19^	13.4	21.5**	6.6**	10.5*

Apical fraction emphysema at -950 HU^†^	0.21 (0-0.72)	9.60 × 10^-30^	0.47**	0.17**	0.16**	0.15*

Airway wall thickness, mm	1.53 (0.25)	5.10 × 10^-54^	1.36**	1.50	1.45**	1.93**

Age (years)	67.4 (6.08)	0.023	66.5	68.6*	66.3*	68.2

BMI (kg/m^2^)	25.1 (3.45)	0.0001	23.7**	25.7*	24.7	26.1*

*Gender (% male)*	*64*	*0.37*	*56*	*64*	*69*	*67*

*Pack-years of smoking*	*67.4 (30.4)*	*0.13*	60.08**	*68.57*	*68.47*	*72.73*

*Age started smoking*	*16.4 (3.58)*	*0.37*	*16.74*	*16.60*	*15.85*	*16.63*

*Age quit smoking*	*57.7 (7.58)*	*0.12*	*56.72*	*58.89*	*56.68*	*58.61*

Pre-bronchodilator FEV_1 _% predicted	25.0 (6.55)	5.80 × 10^-11^	23.48*	28.57**	22.6**	23.89

Pre-bronchodilator FVC % predicted	61.3 (15.4)	4.10 × 10^-7^	59.0	68.2**	57.3**	57.5

Post-bronchodilator FVC % predicted	69.6 (15.7)	6.70 × 10^-14^	67.5	79.1**	62.9**	65.2*

Bronchodilator response, absolute change in FEV_1_, L	0.09 (0.09)	1.90 × 10^-28^	0.08	0.16**	0.04**	0.07**

*FEV_1_/FVC ratio (pre-bronchodilator)*	*0.32 (0.06)*	*0.13*	*0.32*	*0.33*	*0.31*	*0.33*

FEV_1_/FVC ratio (post-bronchodilator)	0.32 (0.06)	2.10 × 10^-5^	0.31	0.34**	0.30**	0.31

Total lung capacity, %predicted	128 (15.3)	3.00 × 10^-4^	132.7**	126.3	129.4	121.2**

Residual volume, % predicted	216 (47.4)	1.20 × 10^-08^	235.3**	195.8**	229.3**	208.9

Diffusion capacity, % predicted	30 (10.1)	4.60 × 10^-6^	25.2**	33.1**	29.0	31.7

Total fraction emphysema at -950 HU^†^	0.15 (0-0.50)	3.40 × 10^-25^	0.31**	0.13**	0.12**	0.11**

Difference between fraction apical and basal emphysema at -950 HU^†^	0.12 (-0.33-0.64)	2.80 × 10^-22^	0.37**	0.08**	0.06**	0.08

Airway wall area, %	73.3 (3.96)	9.90 × 10^-39^	70.4**	73.2	72.4**	78.7**

Square root airway wall area, cm	4.6 (0.50)	2.10 × 10^-32^	4.3**	4.6	4.5**	5.3**

6 minute walk distance (ft)	1265 (318)	1.90 × 10^-5^	1192.2*	1382.1**	1247.2	1155.7*

Maximum work (watts)	43.8 (22.3)	5.20 × 10^-7^	35.5**	53.4**	40.9	40.3

UCSD Shortness of Breath Questionnaire Score	58.9 (17.6)	0.0012	61.4	54.0**	63.5**	57.4

*Arterial pH*	*7.42 (0.03)*	*0.64*	*7.43*	*7.42*	*7.42*	*7.42*

PaO2, mmHg	64.8 (10.8)	0.048	64.2	67.0*	64.3	62.1*

PaCO2, mmHg	42.5 (5.62)	1.00 × 10^-4^	42.0	40.8**	44.3**	43.7

*Exacerbations in year prior to randomization^†^*	*0 (0-4)*	*0.058*	*0*	0**	*0*	*0*

*Exacerbations/year (over 3.3 years)^†^*	*0.16 (0-3.12)*	*0.054*	*0.19*	0.00**	*0.19*	*0.15*

**rs1800470 *TGFB1*^‡^**						

AA	36%	0.04	24%**	42%	34%	45%
		
AG	50%		51%**	46%	55%	57%
		
GG	14%		25%**	12%	12%	8%

Baseline values are for the entire cohort given as mean (sd) unless noted. P values represent tests for groupwise differences between the clusters (see text); values for the clusters represent mean or medians within the cluster. All 31 phenotypic characteristics used for clustering are shown; those not significant at P < 0.05 are displayed in italics. Genotype frequencies are given for the nominally significant association between rs1800470 and cluster assignments.

* P < 0.05, ** P < 0.01 in pairwise comparisons of cluster versus remainder of sample.

† Median (range)

‡ Values given as genotype frequency

Additional phenotypic variables, not included in the cluster analysis, were then examined to determine other characteristics of these clusters. Significant differences among the groups were found for several characteristics (Table 3). Cluster 1, the emphysema predominant cluster, had a lower BMI, fewer pack-years of smoking, higher total lung capacity, and lower diffusing capacity, along with a lower six minute walk distance and maximum work. Consistent with the radiographic clustering and the factor loadings, CT emphysema severity and apical-basal emphysema difference (defined as absolute percent emphysema in the upper lung region minus the lower lung region) were more severe, while airway wall area and square root of wall area were lower. Conversely, cluster 4, the airway predominant cluster, had a higher BMI, lower total lung capacity, and less severe emphysema and higher airway wall measurements, with a lower PaO_2 _and lower six minute walk distance. Cluster 2, the milder severity, bronchodilator responsive subtype, had a higher BMI, greater FVC and DL_CO_, a lower PaCO_2_, higher six minute walk distance and maximum work, fewer symptoms of dyspnea, and fewer exacerbations, despite being of slightly older age. Cluster 3, with a lower FEV_1 _despite less severe radiographic emphysema and airway wall thickness than the other clusters, had more dyspnea and a higher PaCO_2_, with slightly younger age.

To determine whether specific SNPs were associated with cluster membership, we tested genotypes for each of the 12 candidate gene SNPs with cluster membership. A chi-squared P value of 0.034 was seen for a SNP in *TGFB1*, rs1800470; no other P values were nominally (<0.05) significant. In pairwise testing using an additive model of each cluster versus all other clusters, this SNP was associated with membership in cluster 1 (P = 0.002).

Further details on study methods and additional results are available in Additional File 1, including plots of correlations and cluster separation in two-dimensional space (Additional file 1, Figures S1 and S3).

## Discussion

Despite the description of COPD subtypes more than 40 years ago[32] and substantial progress since then in understanding COPD-related phenotypes[33,34], only a few attempts have been made to use statistical methods to define novel COPD subtypes[15,16]. Using a large, well-characterized set of subjects with severe emphysema, we demonstrate the potential utility of using statistical learning methods to find relationships among phenotypic and genotypic characteristics to elucidate disease heterogeneity.

Several methods have attempted to address issues of disease heterogeneity in obstructive airway diseases. Statistical learning techniques such as factor analysis have been used to reveal novel insights into characteristics such as dyspnea or inflammation in COPD[20,35-37]. Cluster analysis has confirmed classic chronic bronchitis and emphysema subtypes[15] or illustrated overlap of characteristics of COPD and asthma[16], and a combination of factor analysis and cluster analysis has defined asthma subtypes[31]. These techniques show promise in identifying disease subtypes (subsets of subjects), or intermediate disease-related phenotypic characteristics (endotypes/endophenotypes[38]). Endophenotypes have already been of substantial utility in genetic association studies in psychiatry[39].

To date, however, there has been limited use of disease subtypes in genetic association studies in COPD. Investigators have tested for specific associations with classic subtypes[11,40,41], or with specific disease-related phenotypic characteristics such as emphysema distribution[22] or functional measures[42]. Factor analysis has been used to demonstrate differences in heritability of components of asthma[43]. Cluster analysis is frequently used in gene expression, and such analyses have been used to define subtypes - though these subtypes have not always been clearly associated with the available clinical characteristics[44]. Our study demonstrates the potential utility of statistical learning methods in the heterogeneous syndrome of COPD.

Our cluster analyses identified four subtypes of subjects in this cohort with severe emphysema: 1) emphysema predominant, 2) milder severity, bronchodilator-responsive, 3) discordant lung function/CT emphysema and airway severity, and 4) airway predominant. Some of the phenotypic associations in these groups, such as a lower BMI with more severe quantitative CT emphysema, have been previously seen[13,45], while others, such as a higher bronchodilator responsiveness in the group with higher FEV_1_, differ from previous reports[46,47]. The association of the nonsynonymous Leu10Pro *TGFB1 *SNP rs1800470 with cluster 1 is consistent with a previously reported association of apical emphysema in this cohort [22] and association of this SNP with reduced lung function has also been seen in a Japanese emphysema cohort[48]. Notably, this SNP has been demonstrated to be of functional significance, with the G allele (C on the reverse strand) resulting in increased production of TGFB1[49]. Several studies have demonstrated an increase in TGFB1 both in the lung[50-52] and in plasma[53] in subjects with COPD, as well as a relationship between TGFB1 levels and lung function, though the relationship between these findings and the rs1800470 genotype is not entirely clear[53].

Conversely, most of the previously reported SNP associations with COPD-related phenotypic characteristics did not demonstrate associations with our clusters. Nonsignificant findings could be due to loss of power from categorical cluster assignment and resulting small sample size, and the use of an omnibus test for genetic association. More importantly, our analysis attempts to determine whether genetic variants lead to a subtype of COPD subjects which share a set of phenotypic characteristics; as such, it does not attempt to determine the specific genotypic-phenotypic variables whose relationship leads to a significant association. Whether one of these approaches - association analysis with individual phenotypic characteristics, or with subtypes of subjects- is superior in identifying replicated genetic associations, or whether the approaches are separately informative, remains to be seen.

Our study has several strengths. First, we used relatively unbiased methods, in both factor analysis and cluster analysis, to select uncorrelated variables and determine severe COPD subtypes using the rich set of phenotypic and quantitative measures available in NETT. Second, our analysis is the largest reported cluster analysis using CT phenotypic variables. Third, despite our homogeneous study population, we were able to discern emphysema subtypes, which differed on variables not used to perform clustering. While all four of these subtypes have not previously been identified, our emphysema and airway-predominant clusters are consistent with a priori defined subtypes used in previous studies[13]. Importantly, recent evidence shows that airway wall thickening and emphysema aggregate independently in families of individuals with COPD[54], suggesting that recognizing these differences may be important for discovering genetic associations.

Our results should be regarded as exploratory for several reasons. First, our dataset was based on available NETT data. Specific relationships between variables - for example, the high correlation between apical and total emphysema - may be due to selection biases of the NETT population. NETT subjects were likely biased towards those without predominant airway disease, and CT scans were suboptimal for assessment of airway wall remodeling due to the thicker slices associated with pre-MDCT (multi-detector CT) imaging. Similarly, our genotypic data was limited to a pre-specified subset of previous positive associations in candidate genes, and our cohort was limited to those enrolled in the NETT Genetics Ancillary Study (Additional File 1, Table S1). Our selection of phenotypic and genotypic variables for inclusion was strongly influenced by the limitations of available data, and decisions were made based on clinical judgement of relevance.

Second, our analysis also found that the separation of clusters was weak, indicating segmentation and not a true separation of these subtypes using clustering. Correspondingly, we found no strong evidence of smaller groups of more distinct subtypes. Furthermore, the small size of our clusters limits the power of association analysis, and our association with rs1800470 was not corrected for multiple comparisons. Given these limitations in this relatively homogeneous cohort, an attempt to validate these findings of specific subtypes using these or similar methods in other well-phenotyped COPD cohorts should be performed. Using a more heterogeneous and less selected group of subjects, in combination with improved radiographic measures, may result in more pronounced and distinct subpopulations.

## Conclusions

The volume of genetic and phenotypic information available in COPD cohorts is rapidly increasing; the number of potential relationships between phenotypic and genotypic characteristics increases exponentially. Statistical learning techniques using multivariate methods, such as dimension reduction and cluster analysis, have the potential to assist in analyses of these complicated problems. Our study demonstrates that application of these techniques, even in a highly selected group of subjects with severe emphysema, has the potential to elucidate phenotypic heterogeneity and disease pathophysiology.

## Competing interests

GJC has received investigational grants from Emphysis Medical Inc, Aeris Therapeutics, Boehringer Ingelheim, Astra Zeneca, GlaxoSmithKline, Forest Pharmaceuticals, and Schering-Plough. EAH is a founder and shareholder of VIDA Diagnostics, Inc. (Coralville, Iowa). EKS has received honoraria from GlaxoSmithKine, Wyeth, Bayer, and Astra-Zeneca, consulting fees from GlaxoSmithKline and Astra-Zeneca, and grant support from GlaxoSmithKline.

## Authors' contributions

MHC carried out the data analysis and drafted the manuscript. EKS conceived and designed the study, and assisted in data analysis and interpretation. GRW and EAH generated the CT data. TH and NL assisted in the statistical analysis. GJC, EAH, and FJM participated in generating the data and in data analysis. JJR helped design the study and assisted in data analysis. All authors read, helped revise, and approved the final manuscript.

## Supplementary Material

Additional file 1 **Supplementary Information**. Supplemental Methods, Results, and Figures. Click here for file
