# Supplementary material for: Cluster analysis in severe emphysema subjects using phenotype and genotype data: an exploratory investigation
Source: Respir Res. 2010 Mar 16;11(1):30. doi: 10.1186/1465-9921-11-30 (PMC2850331; doi:10.1186/1465-9921-11-30)
Supplement: Additional file 1 — Supplementary Information. Supplemental Methods, Results, and Figures. [file 1465-9921-11-30-S1.DOC]

**ONLINE SUPPLEMENT**

Cluster Analysis in Severe Emphysema Subjects Using Phenotype and Genotype Data: An Exploratory Investigation

Michael H. Cho, George R. Washko, Thomas Hoffmann, Gerard J. Criner, Eric A. Hoffman, Fernando J. Martinez, Nan Laird, John J. Reilly, Edwin K. Silverman

**Supplementary Methods**

Chest CT Analysis

Details of the computed tomographic measurements in the NETT Genetics Ancillary Study have been published previously[1]. Briefly, images of the chest were acquired at full inspiration and reconstructed using a high spatial frequency algorithm with a 1 to 2mm collimation at 20mm intervals. Densitometric measures of emphysema were performed using Pulmonary Analysis Software Suite (PASS, Iowa City, IA) [2, 3] and reported as the percentage of lung (%LAA) below a threshold of–950 Hounsfield Units. Airway wall thickness (WT), the square root of wall area (SRWA), and wall area percent (WA%) were assessed using Airway Inspector (www.airwayinspector.org) at Brigham and Women’s Hospital. An average of 12 airways were inspected per subject in the right and left upper lobes and right lower lobe. The full width at half-maximum (FWHM) method was used to measure the WT and SRWA of each airway. From these discrete measures, the WT, SRWA, and WA% (wall area divided by the total cross sectional area of wall and lumen) of a 10mm luminal perimeter airway (Pi10mm) were calculated.

SNP Genotyping

Prior single nucleotide polymorphism (SNP) genotyping data for NETT were generated from one of the following assays: 1) 5’ to 3’ exonuclease TaqMan®[4] method using ABI Pre-Designed SNP Genotyping Assays as per standard protocol, 2) analysis of unlabeled single-base extension minisequencing reactions using the very short extension method on the SEQUENOM MassARRAY MALDI-TOF mass spectrometer (Sequenom, San Diego, CA) with a semiautomated primer design program (SpectroDESIGNER, Sequenom)[5], or 3) allele-specific primer extension (ASPE) gapped ligation assay using the Illumina BeadStation 500G system (Illumina, San Diego, CA)[6].

Statistical Analysis

We calculated Pearson correlation coefficients for all variables (Figure S1) prior to our factor analysis. In our factor analysis, we calculated Bartlett's test of sphericity and the Kaiser-Meyer-Olkin measure of sampling adequacy[7] as a measure of the strength of relationships among variables. We used squared multiple correlations for the prior communality estimates, and we used orthogonal varimax rotation. The number of factors to include was determined by identifying factors explaining at least 75% of the common variance.

We performed cluster analysis using scaled and centered phenotypes. The k-means algorithm chooses a set of random cluster centers. All points are assigned to the nearest center, and the location of that cluster center is adjusted to reflect the mean of the points assigned to that cluster, and this process is repeated. Other algorithms examined were k-medoid, fuzzy clustering, and hierarchical clustering (using Ward’s method). Silhouette width for all clustering analyses was assessed using Euclidean distance and a range of 2 to 6 clusters. Cluster analyses were performed using repeated iterations and random starts until the cluster results were stable.

Group-wise comparisons of each variable among all clusters was performed using a one-way analysis of variance or Kruskal-Wallis test for continuous data, or chi-squared statistic for dichotomous or genetic frequency (SNP) data. Significant (P < 0.05) results were further examined by pairwise comparisons between each cluster and all other clusters using a Wilcoxon or t-test with unequal variances for phenotypic variables, and a Cochran-Armitage test for trend for genetic variables. Two dimension cluster plots were created using principal components analysis to generate x and y axes.

Statistical analyses were performed using R (www.r-project.org) and SAS 9.1 (SAS Institute, Cary, NC). We used factanal in R and PROC FACTOR in SAS to run the factor analysis, and clValid[8] and cluster packages in R for clustering. Pearson correlation coefficients were plotted using the sma package in R.

**Supplementary Results**

The p value for Bartlett's test of sphericity, a test of whether the phenotype correlation matrix is the identity matrix (i.e., no relationship among items) was <0.001, suggesting that factor analysis would be appropriate. The Kaiser-Meyer-Olkin test, a measure of sampling adequacy that compares the magnitude of partial correlation coefficients to the correlation coefficients (ranging from 0 to 1, with higher values indicating stronger evidence of common factors) was 0.56. This result was not unexpected given the variables chosen for inclusion (most of which were not expected to be correlated, or which may have correlation due to an underlying common factor) and the limited heterogeneity of the sample.

Silhouette widths were uniformly higher with k-means than with the other methods; the maximum value for k-medoid clustering was 0.187 at 6 clusters; for fuzzy clustering 0.196 at 2 clusters; and 0.158 for hierarchical clustering at 2 clusters.

**Table S1: Phenotypic Characteristics of NETT Subjects Included Versus Excluded in Cluster Analysis.** Values given as mean (*or median), P value for T-test (*or Wilcoxon Rank Sum) as appropriate. As expected, the included subjects had better pulmonary function and functional capacity than the excluded subjects.

|  | **Excluded from Cluster Analysis**  **N=912** | **Included in Cluster Analysis**  **N=308** | **P value** |
| --- | --- | --- | --- |
| Age, years | 67.05 | 67.40 | 0.384 |
| BMI, kg/m2 | 24.47 | 25.08 | 0.012 |
| Gender (% male) | 64 | 60 | 0.206 |
| Pack years of smoking | 63.70 | 67.43 | 0.069 |
| Age started smoking | 16.87 | 16.42 | 0.075 |
| Age quit smoking | 57.41 | 57.75 | 0.520 |
| Pre-bronchodilator FEV_1_, % predicted | 23.57 | 24.98 | 0.001 |
| Pre-bronchodilator FVC, % predicted | 58.35 | 61.31 | 0.003 |
| Post-bronchodilator FEV_1_, % predicted | 26.24 | 28.28 | 1.63X10^-5^ |
| Post-bronchodilator FVC, % predicted | 66.30 | 69.65 | 0.001 |
| Bronchodilator response, % of baseline FEV_1_ | 0.12 | 0.14 | 0.044 |
| Bronchodilator response, absolute change in FEV_1_, L | 0.077 | 0.095 | 0.001 |
| FEV_1_/FVC ratio, post-bronchodilator | 0.31 | 0.32 | 0.117 |
| FEV_1_/FVC ratio, pre-bronchodilator | 0.32 | 0.32 | 0.475 |
| Total lung capacity, % predicted | 128.39 | 127.72 | 0.504 |
| Residual volume, % predicted | 223.89 | 216.088 | 0.016 |
| Diffusion capacity, % predicted | 27.74 | 30.03 | 3.51x10^-4^ |
| Total fraction emphysema at -950 HU* | 0.133 | 0.147 | 0.153 |
| Difference between apical and basal emphysema at -950 HU* | 0.094 | 0.117 | 0.125 |
| Apical fraction emphysema at -950 HU* | 0.185 | 0.212 | 0.066 |
| Airway wall thickness, mm | NA | 1.53 | 0.998 |
| Airway wall area, % | NA | 73.304 | 0.926 |
| Square root wall area, cm | NA | 4.597 | 0.483 |
| 6 minute walk distance, ft | 1201.80 | 1264.66 | 0.002 |
| Maximum work, watts | 37.44 | 43.78 | 8.15X10^-6^ |
| UCSD Shortness of Breath Questionnaire | 63.70 | 58.88 | 6.43X10^-5^ |
| Arterial pH | 7.42 | 7.42 | 0.574 |
| PaO_2_, mmHg | 64.23 | 64.80 | 0.405 |
| PaCO_2_, mmHg | 43.33 | 42.54 | 0.042 |
| Exacerbations in year prior to randomization | 0.41 | 0.33 | 0.118 |
| Exacerbations / year (over 3.3 years) | 0.30 | 0.27 | 0.195 |

**Figure S1: Correlation matrix between NETT phenotypes:** Pearson correlation coefficients for the 31 NETT phenotypes. Red indicates positive and green indicates negative correlations; intensity indicates degree of correlation.


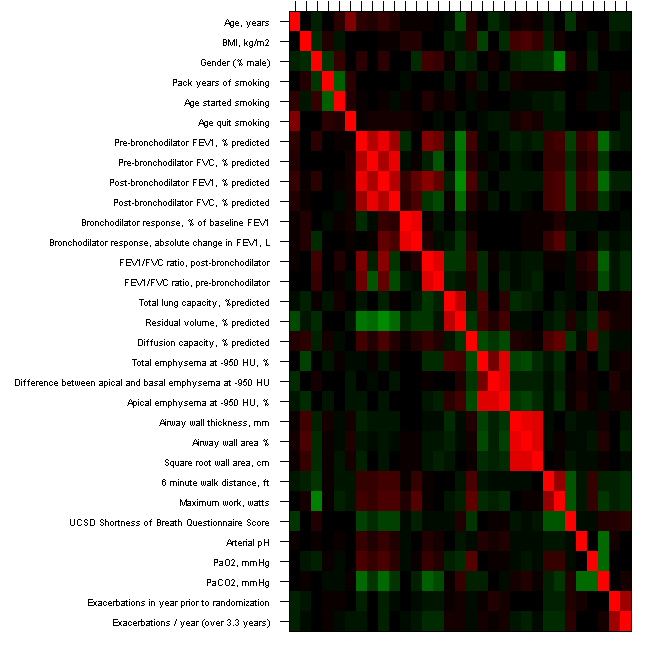


**Figure S2: Pairwise plot of clusters.** Plots of each of the 4 variables selected from factor analysis and used in k-means cluster analysis are shown. Members of each cluster are identified by the symbols and colors indicated by the legend; values of phenotypic variables corresponding to each row and column are given by the x and y-axes.

**
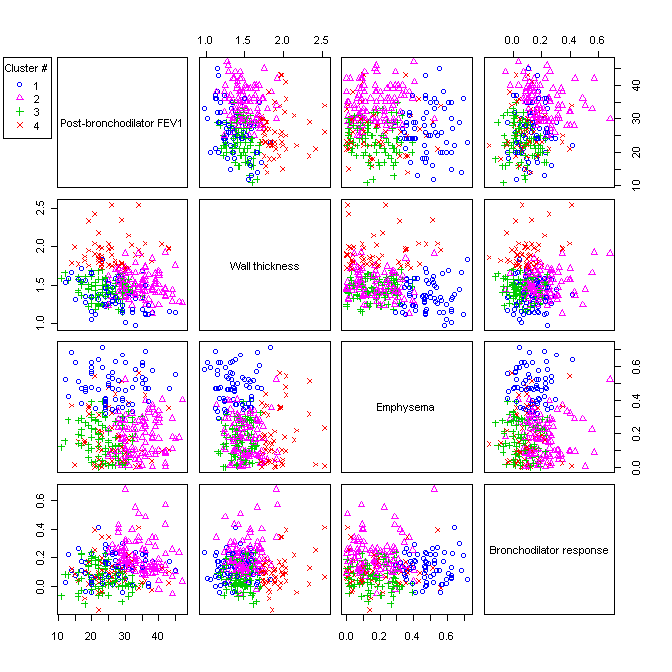
**

**Figure S3: Two dimensional plot of clusters**. To display the subjects in two-dimensional space, principal components were generated from the dataset using the 4 clustering variables; each subject is plotted along the top two principal components (see text).


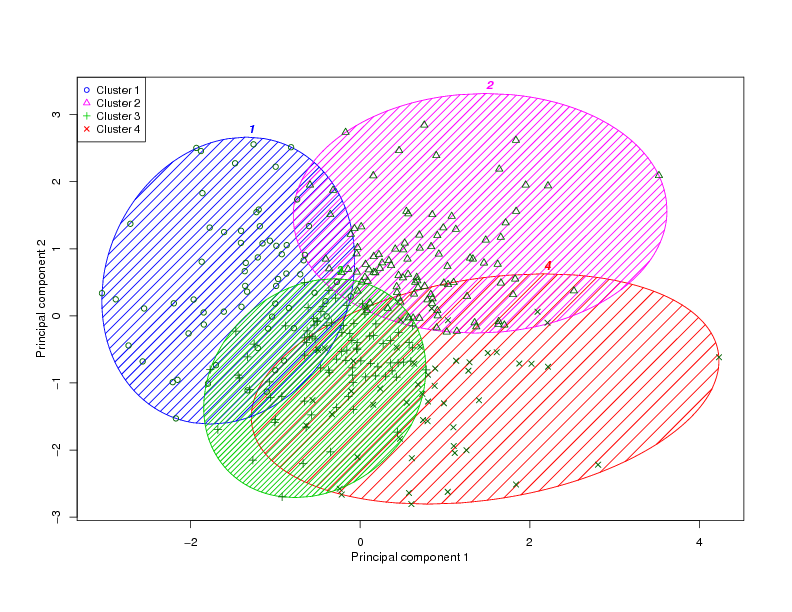


REFERENCES

1. Kim WJ, Silverman EK, Hoffman E, Criner GJ, Mosenifar Z, Sciurba FC, Make BJ, Carey V, Estepar RS, Diaz A *et al*: **CT metrics of airway disease and emphysema in severe COPD**. *Chest* 2009, **136**(2):396-404.

2. Washko GR, Criner GJ, Mohsenifar Z, Sciurba FC, Sharafkhaneh A, Make BJ, Hoffman EA, Reilly JJ: **Computed tomographic-based quantification of emphysema and correlation to pulmonary function and mechanics**. *COPD* 2008, **5**(3):177-186.

3. DeMeo DL, Hersh CP, Hoffman EA, Litonjua AA, Lazarus R, Sparrow D, Benditt JO, Criner G, Make B, Martinez FJ *et al*: **Genetic determinants of emphysema distribution in the National Emphysema Treatment Trial**. *Am J Respir Crit Care Med* 2007, **176**(1):42-48.

4. Holland PM, Abramson RD, Watson R, Gelfand DH: **Detection of specific polymerase chain reaction product by utilizing the 5'----3' exonuclease activity of Thermus aquaticus DNA polymerase**. *Proc Natl Acad Sci U S A* 1991, **88**(16):7276-7280.

5. Tang K, Fu D, Kotter S, Cotter RJ, Cantor CR, Koster H: **Matrix-assisted laser desorption/ionization mass spectrometry of immobilized duplex DNA probes**. *Nucleic Acids Res* 1995, **23**(16):3126-3131.

6. Oliphant A, Barker DL, Stuelpnagel JR, Chee MS: **BeadArray technology: enabling an accurate, cost-effective approach to high-throughput genotyping**. *Biotechniques* 2002, **Suppl**:56-58, 60-51.

7. Pett MA, Lackey NR, Sullivan JJ: **Making sense of factor analysis: the use of factor analysis for instrument development in health care research**. Thousand Oaks, Calif.: Sage Pub.; 2003.

8. Brock G, Pihur V, Datta S, Datta S: **clValid: An R Package for Cluster Validation**. *Journal of Statistical Software* 2008, **25**(4).
